# First Record of *Conotrachelus quadrilineatus* Champion, 1904 (Coleoptera: Curculionidae: Molytinae) Damaging Beans in South America

**DOI:** 10.1007/s13744-024-01187-w

**Published:** 2024-08-19

**Authors:** Daniel A. Baron-Ortiz, Fredy A. Rodriguez-Cruz, Oscar F. Santos-Amaya

**Affiliations:** 1 Federación Nacional de Cultivadores de Cereales, Leguminosas y Soya, Fenalce, Yopal, Colombia; 2 Dept of Agronomy, Univ de los Llanos, Villavicencio, Colombia; 3 Dept of Agronomy, Univ de Pamplona, Pamplona, Colombia

**Keywords:** *Phaseolus vulgaris*, *Conotrachelus*, Pest, Bean borer

## Abstract

Common bean (*Phaseolus vulgaris* L.) is the most important legume used for direct human consumption in Latin America, with an increasing expansion of cultivated areas in recent years. Here, we report the first occurrence of *Conotrachelus quadrilineatus* Champion, 1904 (Coleoptera: Curculionidae: Molytinae) feeding on bean in South America. Larvae and adults of *C. quadrilineatus* were observed during the first half of 2022, severely affecting the plantations of 20 bean farmers in the municipality of Garcia-Rovira, Santander Colombia. It is necessary to describe and quantify the damage of *C. quadrilineatus* in bean crops, as well as to study its bioecology.

Common bean (*Phaseolus vulgaris* L.) is the most important legume after maize and wheat for human consumption (Uebersax et al. 2023), being a staple food in Latin America and East Africa and Asia (FAO, 2023). In these regions, beans are grown mainly by small-scale farmers, which produce about 77% of the world production (Uebersax et al. 2023). Beans play an important role in food security and combating malnutrition in the world. In South America, beans are grown in Argentina, Bolivia, Chile, Colombia, Ecuador, Paraguay, Peru, Uruguay, Venezuela, and Brazil (Siddiq et al. 2022).

One of the main limitations to global food production is the losses caused by arthropod pests (Savary et al. 2019). In January 2022, we received reports of injuries in bean crops caused by beetle larvae that were boring into the stems of the plants in the municipality of Garcia-Rovira, Santander Colombia, something that had not been reported to date in this crop in South America. Therefore, the objective of this research was to identify and report the occurrence of phytophagous beetle larvae that were affecting *P. vulgaris* plants in Colombia.

In the company of farmers, we took larvae that were inside the stems of bean plants. This was carried out in 20 fields planted with beans in the municipality of Garcia-Rovira, Santander, Colombia (Fig. 1). Approximately 20 larvae from each planted plantation were taken to the Agricultural Entomology Laboratory of the University of Pamplona, where they were kept under controlled conditions of temperature and relative humidity (24 ± 1°C; 70 ± 5% rh) until the emergence of the adults. Twenty specimens were sent to Dr. Robert S. Anderson (specialist in Systematics, phylogeny, and biology of Curculionoidea with a special emphasis on New World taxa, from the Canadian Museum of Nature). The specimies were identified as *Conotrachelus quadrilineatus* Champion, 1904 (Coleoptera: Curculionidae: Molytinae) (Fig. 2).

**Fig. 1:**
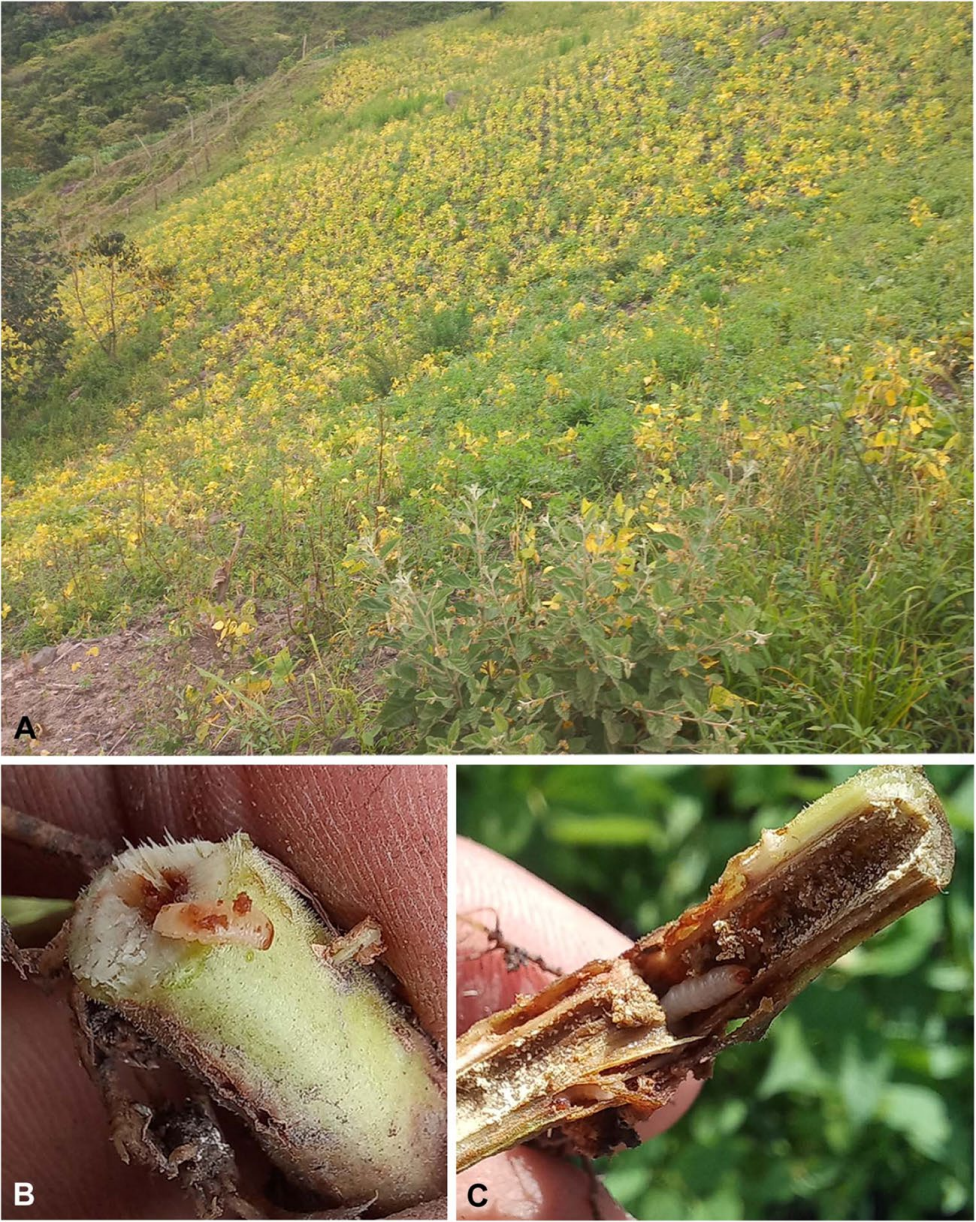
Bean crops attacked by *Conotrachelus quadrilineatus* Champion, 1904 (Coleoptera: Curculionidae: Molytinae) in the municipality of Garcia-Rovira, Santander, Colombia. **A** Chlorotic plants affected by *Conotrachelus quadrilineatus*.; **B**, **C** *Conotrachelus quadrilineatus* larvae boring into bean stems

**Fig. 2:**
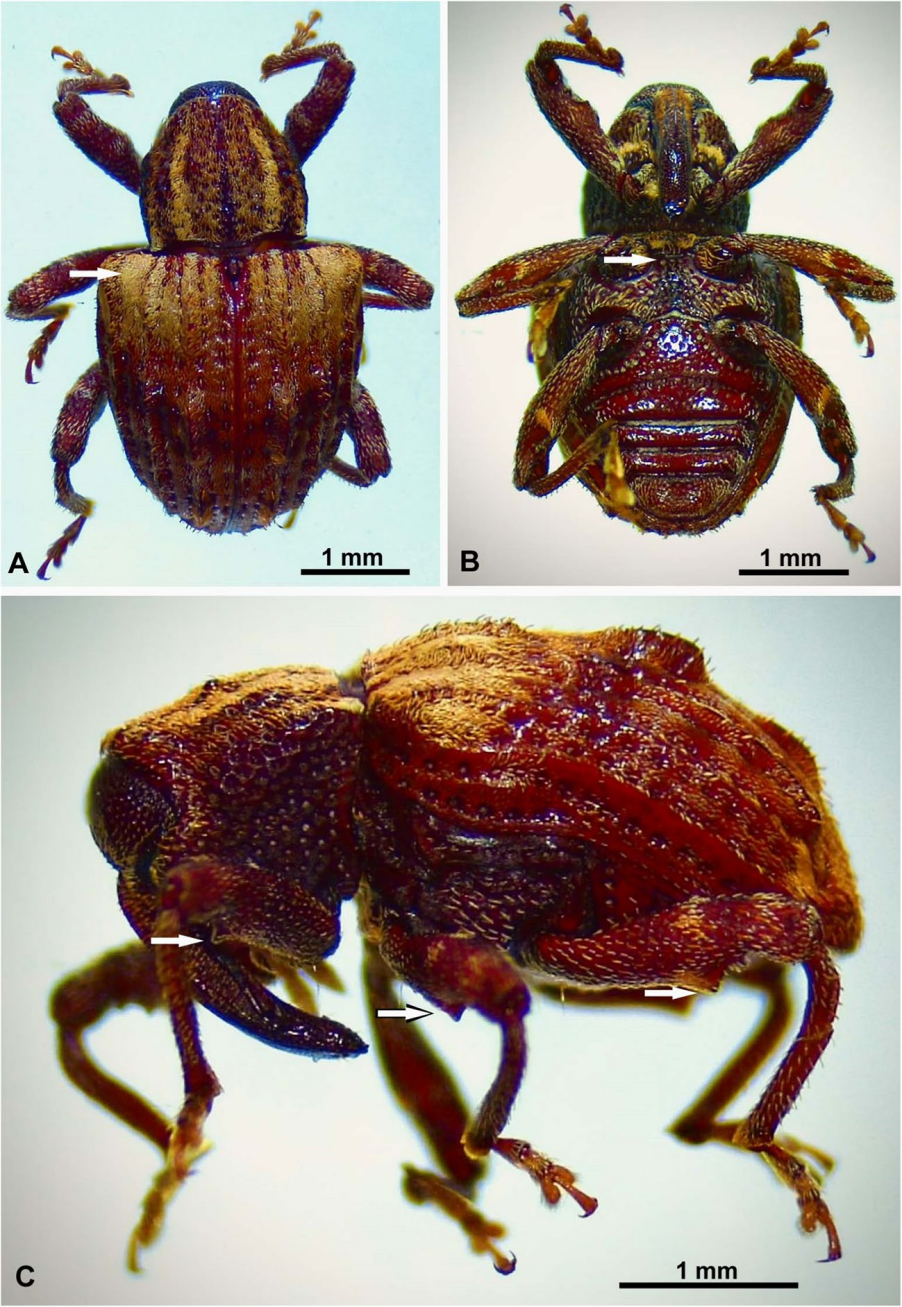
*Conotrachelus quadrilineatus* adults. **A** Dorsal view, note the vestiture in the angles of the elytron (arrow) (**B**); ventral view, note the mesosternum flattened between coxae (arrow) (**C**); lateral view, note the femora clavate, each with a triangular tooth (arrows). Scale bars = 1.0 mm

Although the genus *Conotrachelus* Dejean, 1835 (Coleoptera, Curculionidae) is considered the most diverse genus of weevil in the Americas (O’Brien & Couturier 1995), with more than 1200 species currently identified (Pinzón-Navarro et al. 2010; Castañeda-Vildózola et al. 2014; Anderson 2021), this is the first report from *C. quadrilineatus* in South America based on the available literature. The only record of a specimen in the gender was reported in Panama (Champions, 1902). The large number of individuals found in the field reported in this study suggest that *C. quadrilineatus* might be present in other producing regions in Colombia.

Species of the genus *Conotrachelus* are important agricultural pests in the Neotropical region, because they feed on the reproductive and vegetative structures of a broad diversity of dicotyledons (*Persea americana* Mill, *Psidium guajava* L, *Crataegus* spp., *Malphigia mexicana* Juss.) (Marvaldi et al. 2002). In Colombia, the main species of this genus is *C. psidii* Marshall, which attacks guava crops in all regions of the country (Monroy & Insuasty 2006). To our knowledge, no other species of this genus has been reported attacking bean crops. Therefore, this is the first report of a species of this genus feeding on beans in Colombia and South America, this being the first record of this species causing damage to an important crop in this region of the world.

The identification of insect species in agriculture is necessary for decision-making when implementing a pest management program (Deguine et al. 2021), or, in the case of quarantine pests, avoiding the closure of markets due to an incorrect determination (EFSA et al. 2018). With the identification of *C. quadrilineatus* attacking beans, the first step is completed. However, it is necessary to describe and quantify the damage of *C. quadrilineatus* in this crop, as well as to study its bioecology.

This is the first report of *C. quadrilineatus* in South America, as well as the attack of its larvae on bean crops in this region. The high incidence in the sampled area and the significant lesions observed indicate that this species might pose a great threat to beans production in Colombia. Research should be carried out to assess whether this is an isolated case or whether *C. quadrilineatus* is already spread across bean-producing regions of this country.

## Declarations

This article does not contain any studies with human participants or animals performed by any of the authors.
